# Procalcitonin metabolomics in the critically ill reveal relationships between inflammation intensity and energy utilization pathways

**DOI:** 10.1038/s41598-021-02679-0

**Published:** 2021-12-01

**Authors:** Hirotada Kobayashi, Karin Amrein, Jessica A. Lasky-Su, Kenneth B. Christopher

**Affiliations:** 1 Division of Renal Medicine, Brigham and Women’s Hospital, Harvard Medical School, Boston, USA; 2 Division of Endocrinology and Diabetology, Medical University of Graz, Graz, Austria; 3 Channing Division of Network Medicine, Brigham and Women’s Hospital, Boston, USA; 4 Division of Renal Medicine, Channing Division of Network Medicine, Brigham and Women’s Hospital, 75 Francis Street, Boston, MA 02115 USA

**Keywords:** Bioenergetics, Metabolomics, Randomized controlled trials, Acute inflammation

## Abstract

Procalcitonin is a biomarker of systemic inflammation and may have importance in the immune response. The metabolic response to elevated procalcitonin in critical illness is not known. The response to inflammation is vitally important to understanding metabolism alterations during extreme stress. Our aim was to determine if patients with elevated procalcitonin have differences in the metabolomic response to early critical illness. We performed a metabolomics study of the VITdAL-ICU trial where subjects received high dose vitamin D_3_ or placebo. Mixed-effects modeling was used to study changes in metabolites over time relative to procalcitonin levels adjusted for age, Simplified Acute Physiology Score II, admission diagnosis, day 0 25-hydroxyvitamin D level, and the 25-hydroxyvitamin D response to intervention. With elevated procalcitonin, multiple members of the short and medium chain acylcarnitine, dicarboxylate fatty acid, branched-chain amino acid, and pentose phosphate pathway metabolite classes had significantly positive false discovery rate corrected associations. Further, multiple long chain acylcarnitines and lysophosphatidylcholines had significantly negative false discovery rate corrected associations with elevated procalcitonin. Gaussian graphical model analysis revealed functional modules specific to elevated procalcitonin. Our findings show that metabolite differences exist with increased procalcitonin indicating activation of branched chain amino acid dehydrogenase and a metabolic shift.

## Introduction

Procalcitonin is a 116-amino acid polypeptide detectable in the blood of healthy adults that rapidly increases 1000-fold with severe critical illness^1,2^. The release of procalcitonin from parenchymal tissue into circulation is stimulated by microbial toxins and modulated by the immune response^3,4^. Procalcitonin is a commonly used biomarker of severity of systemic inflammation in infection, sepsis, trauma, surgery, cardiogenic shock, autoimmune disease and severe COVID-19^5,6^. Although published data suggested that procalcitonin has a greater role in inflammation than previously thought, the metabolic response to procalcitonin has not been evaluated^7–11^. Examining the role of procalcitonin in the metabolic response to inflammation can provide a more nuanced understanding of the immune system during the extreme stress of early critical illness.

Procalcitonin may have a detrimental role in the host response to inflammation in critical illness. Procalcitonin is shown to enhance the inflammatory response and stimulate the surface expression of CD16 on human neutrophils and CD14 on lymphocytes^7,8^. In experimental studies, procalcitonin exposure leads to endothelial barrier function impairment and hepatocyte dysfunction^9,10^. Further, experimental models of sepsis show the presence of procalcitonin worsens illness severity and outcomes^11^.

Metabolomic studies performed on blood collected early in critical illness show a profound disturbance of metabolic homeostasis that reflects illness severity and is predictive of adverse outcomes^12^. But such work has not addressed the metabolic response to inflammation^13^. Therefore, we performed a cohort study on the associations between increased procalcitonin levels and changes in metabolites during critical illness. We used global metabolomic profiling to capture a diverse range of metabolites that are measured in plasma, reflecting multiple metabolism pathways. We hypothesize that there is a specific metabolomic profile that represents a response to elevated procalcitonin in critical illness. We measured the abundance of 983 metabolites from 1187 plasma samples over three time points in 419 critically ill subjects collected during the VITdAL-ICU trial^14,15^. We determined the effect of increased procalcitonin on changes in individual metabolites and metabolic pathways over time. Further, we analyzed grouping of specific functionally related metabolites that change in unison with increased procalcitonin.

## Results

In the analytic cohort (N = 419), we found the median [interquartile range] of procalcitonin at day 0 was 0.66 [0.17, 2.79] µg/L, at day 3 was 0.35 [0.12, 1.43] µg/L and at day 7 was 0.21 [0.09, 0.73] µg/L. Baseline characteristics of the cohort were balanced between subjects grouped by procalcitonin level for age, SAPS II score, 25(OH)D level at day 0, intervention status and the absolute change in 25(OH)D level at day 3. Differences existed with respect to sex, C-reactive protein, Day 0 total bilirubin and creatinine, ICU type, and admission diagnosis category (Table 1, Supplementary Table S1). The overall 28-day mortality of the 419 subject analytic cohort was 22.6%.

**Table 1:** Analytic cohort characteristics by Day 0 procalcitonin levels.

Characteristic	Day 0 procalcitonin		Total	P-value
	< 0.50 µg/L	≥ 0.50 µg/L		
No	180	239	419	
Age years Mean (SD)	63.1 (15.5)	65.4 (14.2)	64.4 (14.8)	0.11
Female No. (%)	76 (42)	73 (31)	149 (36)	0.013
SAPS II Mean (SD)	32.3 (16.5)	34.2 (14.5)	33.4 (15.4)	0.22
C-reactive protein Day 0 Mean (SD)	87.0 (74.5)	152.3 (90.8)	124.2 (90.1)	< 0.001
Day 0 25(OH)D Mean (SD)	14.6 (6.3)	13.4 (10.4)	13.9 (8.9)	0.19
Vitamin D_3_ Intervention No. (%)	79 (44)	127 (53)	205 (49)	0.061
Change in 25(OH)D Day 0 to Day 3 Median [IQR]	2.8 [− 0.4, 25.1]	3.3 [0.1, 12.2]	3.1 [0, 16.7]	0.34
Total Bilirubin Day 0 Mean (SD)	0.8 (0.9)	2.1 (3.3)	1.6 (2.6)	< 0.001
Creatinine Day 0 Mean (SD)	1.0 (0.7)	1.7 (1.1)	1.4 (1.0)	< 0.001
**ICU**				< 0.001
Anesthesia ICU No. (%)	27 (15)	53 (22)	80 (19)	
Cardiac surgery ICU No. (%)	27 (15)	95 (40)	122 (29)	
Medical ICU No. (%)	29 (16)	60 (25)	89 (21)	
Neurological ICU No. (%)	88 (49)	18 (8)	106 (25)	
Surgical ICU No. (%)	9 (5)	13 (5)	22 (5)	
28-day mortality No. (%)	25 (14)	70 (29)	95 (23)	< 0.001

### Single time point data

We utilized mass spectrometry methods by Metabolon, Inc to investigate circulating changes in metabolites associated with increased procalcitonin. In day 0 plasma samples (N = 419), significant crude differences exist in 591 individual metabolites (q-value threshold of 0.05) in subjects with or without procalcitonin ≥ 0.5 μg/L notable for increases of branched-chain amino acids (BCAAs), short and medium chain acylcarnitines, dicarboxylate fatty acid pathways and decreases in long-chain acylcarnitines, lysophosphatidylcholine and sphingomyelin metabolites (Supplementary Data S1). Regarding differences in metabolomic profiles of subjects with or without procalcitonin ≥ 0.5 μg/L at day 0, the OPLS-DA model had acceptable predictability (Q2 value 0.427). Confirmation of the stability and robustness of the OPLS-DA model was shown by the permutation test (Q2 intercept of − 0.214, p-value ≤ 0.05) with a negative permutation Q2 intercept indicating model validity (Supplementary Table S2). The cross-validation procedure showed that the groups with or without procalcitonin ≥ 0.5 μg/L were significantly separated (CV-ANOVA p-value < 0.001). The ROC analysis showed the predictive ability of the OPLS-DA model was excellent (AUC = 0.92). Further, the model showed good classification performance with 83.1% of cases with procalcitonin ≥ 0.5 μg/L were correctly classified (sensitivity of 85.6%, specificity of 79.8%).

### Multiple time point data

**Figure 1:**
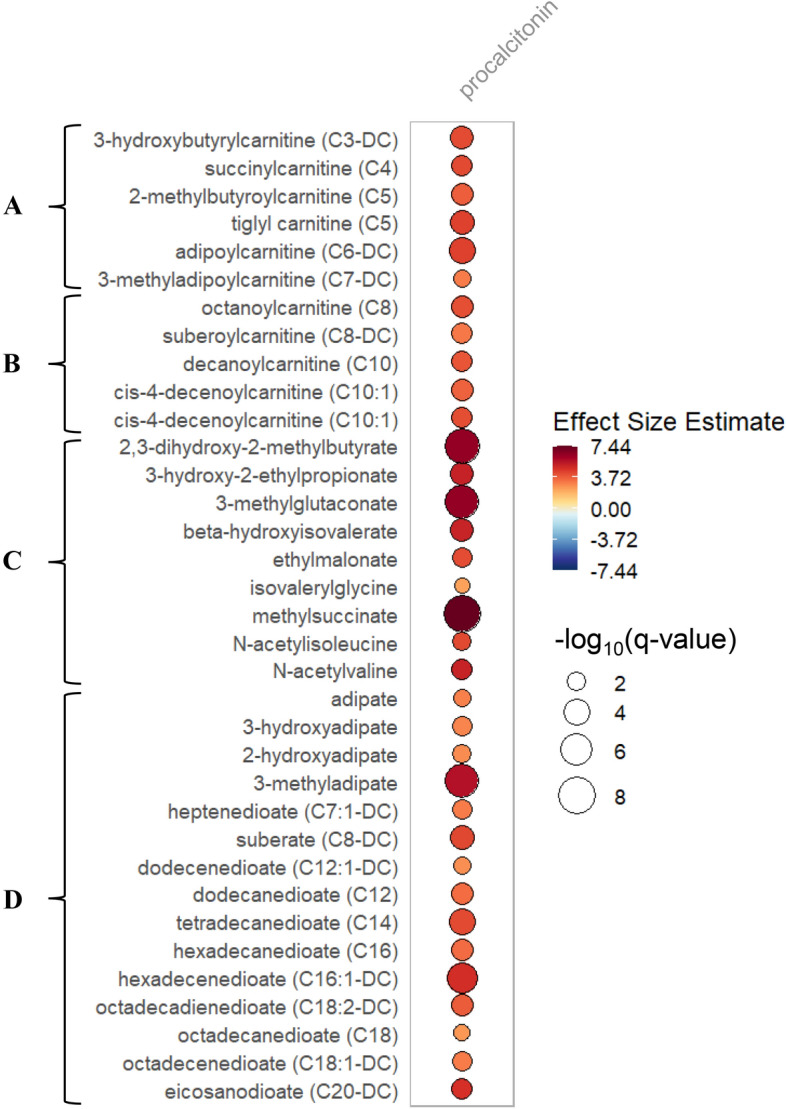
Rain plot of metabolites significantly increased with increased Procalcitonin. Repeated measures metabolomics data (day 0, 3 and 7) relative to procalcitonin level. Correlations between procalcitonin levels and individual metabolite abundance at day 0, 3 or 7 were determined utilizing linear regression models correcting for age, sex, SAPS II, admission diagnosis, 25(OH)D at day 0 and for absolute change in 25(OH)D level at day 3. The magnitude of beta coefficient estimates is shown by a color fill scale and the corresponding significance level (− log10(q-value)) is represented by size of the circle. The intensity of the red fill color represents an increase in effect size for that metabolite relative to procalcitonin level. All metabolites shown are significant by a q-value threshold of 0.05. All respective β coefficients and q-values can be found in tabular form in Supplementary Data S3. (**A**) Short-chain acylcarnitines (**B**) Medium-chain acylcarnitines (**C**) BCAA metabolites (**D**) Dicarboxylate fatty acids.

**Figure 2:**
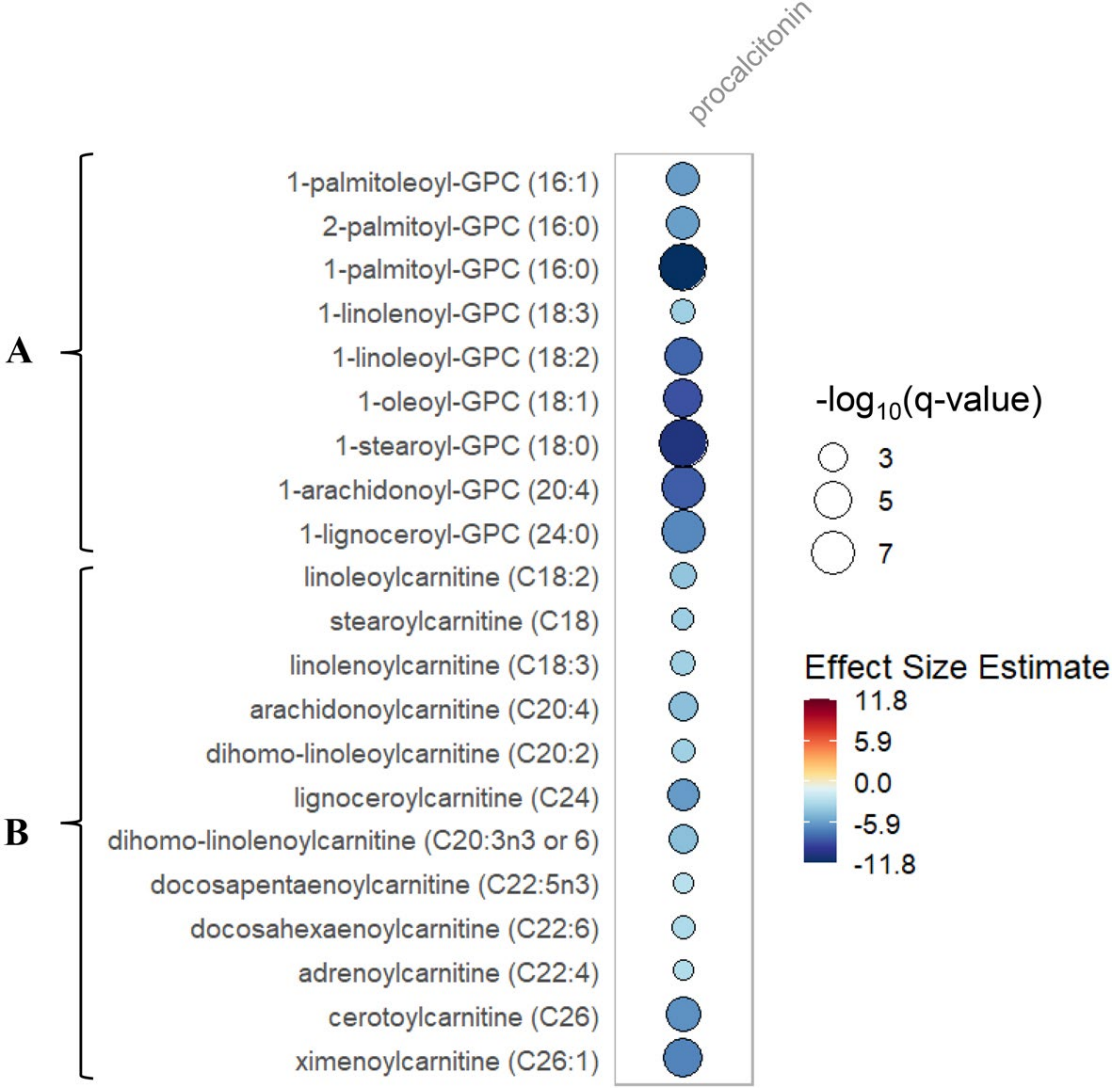
Rain plot of metabolites significantly decreased with increased Procalcitonin. Correlations between procalcitonin levels and individual metabolite abundance at day 0, 3 or 7 were determined utilizing linear regression models correcting for age, SAPS II, admission diagnosis, 25(OH)D at day 0 and for absolute change in 25(OH)D level at day 3. The magnitude of beta coefficient estimates is shown by a color fill scale and the corresponding significance level (− log_10_(q-value)) is represented by size of the circle. The intensity of the blue fill color represents a decrease in effect size for that metabolite relative to procalcitonin level. All metabolites shown are significant by a q-value threshold of 0.05. All respective β coefficients and q-values can be found in tabular form in Supplementary Data S3. (**A**) Lysophosphatidylcholines (**B**) Long chain acylcarnitines.

In the repeated measure metabolomics data, mixed-effects modeling of 1187 plasma samples collected at day 0, 3 and 7 from 419 VITdAL-ICU trial subjects (Model 1), 250 metabolites had significantly positive associations with procalcitonin. The metabolites were dominated by increases in BCAAs, short and medium-chain acylcarnitines, dicarboxylate fatty acids, phosphatidylethanolamines, and polyamines (Table 2, Fig. 1, Supplementary Data S2). One-hundred nineteen metabolites had a significant negative association with procalcitonin, including multiple representatives of the lysophosphatidylcholine, and long-chain acylcarnitine pathway metabolites (Table 3, Fig. 2, Supplementary Data S2). The rain plots show highlighted metabolites that are significantly increased (Fig. 1) or decreased (Fig. 2) in subjects with increased procalcitonin. Similar metabolite patterns were observed in mixed effects models restricted to the 603 day 0, 3 and 7 samples from 213 subjects who received placebo (Supplementary Data S3). A q-value threshold of 0.05 was used to identify all significant mixed effects associations^16^.

**Table 2:** Metabolites significantly increased with increased Procalcitonin over days 0–7.

Metabolite	β coefficient metabolite	p-value	− log10p	q-value	Super pathway	Sub pathway
3-Hydroxybutyrylcarnitine (C3-DC)	4.17	1.19 E−04	3.92	6.78 E−04	Lipid	Short-chain acylcarnitine
Succinylcarnitine (C4)	4.22	7.58 E−04	3.12	3.17 E−03	Energy	Short-chain acylcarnitine
Tiglyl carnitine (C5)	4.42	9.86 E−05	4.01	5.89 E−04	Amino acid	Short-chain acylcarnitine
2-Methylbutyroylcarnitine (C5)	3.73	4.04 E−04	3.39	1.86 E−03	Amino acid	Short-chain acylcarnitine
Adipoylcarnitine (C6-DC)	4.42	1.50 E−05	4.82	1.20 E−04	Lipid	Short-chain acylcarnitine
3-Methyladipoylcarnitine (C7-DC)	3.08	4.32 E−03	2.36	1.45 E−02	Lipid	Short-chain acylcarnitine
Octanoylcarnitine (C8)	4.09	3.83 E−04	3.42	1.79 E−03	Lipid	Medium-chain acylcarnitine
Suberoylcarnitine (C8-DC)	3.19	5.57 E–−04	3.25	2.45 E−03	Lipid	Medium-chain acylcarnitine
cis-4-Decenoylcarnitine (C10:1)	4.12	7.12 E−04	3.15	2.99 E−03	Lipid	Medium-chain acylcarnitine
Decanoylcarnitine (C10)	3.94	6.74 E−04	3.17	2.86 E−03	Lipid	Medium-chain Acylcarnitine
3-Methyladipate	5.74	7.40 E−09	8.13	1.91 E−07	Lipid	Fatty acid, dicarboxylate
Adipate	3.00	4.31 E−03	2.37	1.45 E−02	Lipid	Fatty acid, dicarboxylate
3-Hydroxyadipate^a^	2.91	1.89 E−03	2.72	7.03 E−03	Lipid	Fatty acid, dicarboxylate
2-Hydroxyadipate	2.77	3.30 E−03	2.48	1.15 E−02	Lipid	Fatty acid, dicarboxylate
heptenedioate (C7:1-DC)^a^	3.08	1.83 E−03	2.74	6.83 E−03	Lipid	Fatty acid, dicarboxylate
Suberate (C8-DC)	4.26	9.47 E−05	4.02	5.80 E−04	Lipid	Fatty acid, dicarboxylate
Dodecanedioate (C12)	3.41	3.01 E−04	3.52	1.46 E–03	Lipid	Fatty acid, dicarboxylate
Dodecenedioate (C12:1-DC)^a^	2.69	4.47 E−03	2.35	1.50 E−02	Lipid	Fatty acid, dicarboxylate
Hexadecanedioate (C16)	3.43	2.75 E−04	3.56	1.35 E−03	Lipid	Fatty acid, dicarboxylate
Octadecadienedioate (C18:2-DC)^a^	3.81	2.86 E−04	3.54	1.40 E−03	Lipid	Fatty acid, dicarboxylate
Octadecenedioate (C18:1-DC)^a^	3.09	1.50 E−03	2.83	5.82 E−03	Lipid	Fatty acid, dicarboxylate
Octadecanedioate (C18)	2.51	7.54 E−03	2.12	2.32 E−02	Lipid	Fatty acid, dicarboxylate
Eicosanodioate (C20-DC)	4.77	6.57 E−04	3.18	2.80 E−03	Lipid	Fatty acid, dicarboxylate
Methylsuccinate	7.44	1.87 E−10	9.73	8.31 E−09	Amino acid	BCAA metabolism
3-Methylglutaconate	6.46	7.79 E−09	8.11	1.96 E−07	Amino acid	BCAA metabolism
2,3-Dihydroxy-2-methylbutyrate	6.44	2.75 E−09	8.56	8.70 E−08	Amino acid	BCAA metabolism
3-Hydroxy-2-ethylpropionate	5.22	1.64 E−04	3.79	8.72 E−04	Amino acid	BCAA metabolism
*N*-Acetylvaline	5.19	6.79 E−04	3.17	2.87 E−03	Amino acid	BCAA metabolism
beta-Hydroxyisovalerate	5.16	1.30 E−04	3.89	7.20 E−04	Amino acid	BCAA metabolism
*N*-Acetylisoleucine	4.25	2.35 E−03	2.63	8.50 E−03	Amino acid	BCAA metabolism
Ethylmalonate	4.16	1.29 E−03	2.89	5.21 E−03	Amino acid	BCAA metabolism
Isovalerylglycine	2.29	1.52 E−02	1.82	4.08 E−02	Amino acid	BCAA metabolism
Sedoheptulose	4.78	2.40 E−05	4.62	1.83 E−04	Carbohydrate	Pentose metabolism
Arabonate/xylonate	4.37	5.22 E−04	3.28	2.35 E−03	Carbohydrate	Pentose metabolism
Ribonate	3.98	3.03 E−03	2.52	1.07 E−02	Carbohydrate	Pentose metabolism
Arabinose	3.60	1.31 E−03	2.88	5.26 E−03	Carbohydrate	Pentose metabolism
Ribitol	3.26	1.30 E−02	1.89	3.61 E−02	Carbohydrate	Pentose metabolism

Using repeated measures data (day 0, 3 and 7), the association between relative quantitation of each individual metabolite noted above and Procalcitonin levels over time were determined utilizing linear mixed-effects models correcting for age, sex, baseline 25(OH)D, absolute increase in 25(OH)D, SAPS II, admission diagnosis, plasma day and an individual subject-specific random-intercept. All significant mixed-effects associations have false discovery rate adjusted p-value (q-value) < 0.05. BCAA is Branched-Chain Amino Acids inclusive of Leucine, Isoleucine and Valine. For the Acylcarnitines sub pathway: a capital C is followed by the number of carbons within the fatty acyl group attached to the carnitine. A colon followed by a number is one or more unsaturated carbons in the acylcarnitine ester (i.e. C10:1 is a monounsaturated C10 acylcarnitine). DC following the carbon number is a dicarboxylic acylcarnitine.

^a^Putative identification (Level 2) where predictive or externally acquired structure evidence is present when a reference standard does not exist.

**Table 3:** Metabolites significantly decreased with increased Procalcitonin over days 0–7.

Metabolite	β coefficient metabolite	p-value	− log10p	q-value	Super pathway	Sub pathway
Linolenoylcarnitine (C18:3)*	− 3.58	1.65 E−03	2.78	6.26 E−03	Lipid	Long-chain acylcarnitine
Stearoylcarnitine (C18)	− 3.68	5.40 E−03	2.27	1.76 E−02	Lipid	Long-chain acylcarnitine
Linoleoylcarnitine (C18:2)*	− 4.16	9.56 E−04	3.02	3.94 E−03	Lipid	Long-chain acylcarnitine
Arachidonoylcarnitine (C20:4)	− 4.41	1.57 E−04	3.80	8.47 E−04	Lipid	Long-chain acylcarnitine
Dihomo-linoleoylcarnitine (C20:2)*	− 3.62	2.78 E−03	2.56	9.98 E−03	Lipid	Long-chain acylcarnitine
Dihomo-linolenoylcarnitine (C20:3n3 or 6)*	− 4.43	2.13 E−04	3.67	1.09 E−03	Lipid	Long-chain acylcarnitine
Lignoceroylcarnitine (C24)*	− 6.03	3.93 E−05	4.41	2.75 E−04	Lipid	Long-chain acylcarnitine
Docosapentaenoylcarnitine (C22:5n3)*	− 2.67	1.12 E−02	1.95	3.25 E−02	Lipid	Long-chain acylcarnitine
Adrenoylcarnitine (C22:4)*	− 2.89	9.64 E−03	2.02	2.84 E−02	Lipid	Long-chain acylcarnitine
Docosahexaenoylcarnitine (C22:6)*	− 3.07	3.79 E−03	2.42	1.29 E−02	Lipid	Long-chain acylcarnitine
Cerotoylcarnitine (C26)*	− 6.43	3.43 E−06	5.47	3.69 E−05	Lipid	Long-chain acylcarnitine
Ximenoylcarnitine (C26:1)*	− 6.92	1.12 E−07	6.95	1.96 E−06	Lipid	Long-chain acylcarnitine
2-Palmitoyl-GPC* (16:0)*	− 5.86	8.40 E−06	5.08	7.35 E−05	Lipid	Lysophosphatidylcholine
1-Palmitoleoyl-GPC* (16:1)*	− 5.95	1.37 E−05	4.86	1.12 E−04	Lipid	Lysophosphatidylcholine
1-Palmitoyl-GPC (16:0)	− 11.80	6.98 E−11	10.16	3.26 E−09	Lipid	Lysophosphatidylcholine
1-Linolenoyl-GPC (18:3)*	− 3.67	1.49 E−03	2.83	5.82 E−03	Lipid	Lysophosphatidylcholine
1-Linoleoyl-GPC (18:2)	− 7.97	6.65 E−07	6.18	9.17 E−06	Lipid	Lysophosphatidylcholine
1-Oleoyl-GPC (18:1)	− 8.74	2.17 E−07	6.66	3.48 E−06	Lipid	Lysophosphatidylcholine
1-Lignoceroyl-GPC (24:0)	− 6.74	3.39 E−09	8.47	1.02 E−07	Lipid	Lysophosphatidylcholine

Using repeated measures data (day 0, 3 and 7), the association between relative quantitation of each individual metabolite noted above and Procalcitonin levels over time were determined utilizing linear mixed-effects models correcting for age, sex, baseline 25(OH)D, absolute increase in 25(OH)D, SAPS II, admission diagnosis, plasma day and an individual subject-specific random-intercept. All significant mixed-effects associations have false discovery rate adjusted p-value (q-value) < 0.05. For the Acylcarnitines sub pathway: a capital C is followed by the number of carbons within the fatty acyl group attached to the carnitine. A colon followed by a number is one or more unsaturated carbons in the acylcarnitine ester (i.e. C26:1 is a monounsaturated C26 acylcarnitine). GPC is glycerylphosphorylcholine.

### Metabolic networks and mediation

We next explored procalcitonin-specific relationships between metabolites. With Gaussian graphical models (GGMs) we measured pairwise correlations in metabolites that have similar effects. The GGM analyses revealed six procalcitonin-specific functional modules at day 0 (Supplementary Table S3). Similar to the mixed effects analyses (Model 1), metabolism of acylcarnitines, polyamines, BCAAs, and dicarboxylate fatty acids are prominently featured in the procalcitonin-specific GGM modules. Metabolites within in each functional module were increased with increased procalcitonin in unison as well as having biological and functional similarity (i.e., in Supplementary Table S3 Module C, 5 of 6 members are polyamide metabolites).

To determine the potential mediation of the relationship between metabolite abundance and procalcitonin levels we focused on liver function, body mass index and age. Mediation analyses in day 0 data revealed no influence of body mass index or age on associations between procalcitonin levels and all 983 metabolites. With regard to bilirubin, mediation analyses in day 0 data revealed a significant influence on associations between procalcitonin levels and bilirubin of 171 of the 983 individual metabolites (all p-values were < 0.01 and proportion mediated over 10% using 2000 bootstrap samples). One hundred fifty-seven of these mediated metabolites were also identified in our mixed-effects analysis as significantly changed with procalcitonin levels (Supplementary Data S4).

Finally, to explore mechanistic insights into our observation of increased BCAA metabolites with increasing procalcitonin, we present unadjusted metabolite abundance data at day 0, 3 and 7 for subjects with procalcitonin < 0.5 μg/L or ≥ 0.5 μg/L (Supplementary Fig. S1). Boxplots show relative abundance of plasma BCAAs, branched-chain keto acids, metabolites downstream from branched-chain amino acid dehydrogenase (BCKDH) and BCAA-derived carnitines. The abundance of BCAA metabolites downstream from the BCKDH enzyme complex increase at days 3 and 7 in subjects with procalcitonin ≥ 0.5 μg/L but not with procalcitonin < 0.5 μg/L (Supplementary Fig. S1J–T).

## Discussion

The underlying mechanisms of alterations of metabolomic disruption during inflammation remain an enigma. Using multiple analytic approaches, our metabolomics study detected groups of metabolites along similar sub-pathways with strong associations with procalcitonin levels. In the setting of an elevated procalcitonin, our data highlight increases in short-chain acylcarnitines, dicarboxylate fatty acids, phosphatidylethanolamines, and polyamines and decreases in long-chain acylcarnitines, lysophosphatidylcholines, lysoplasmalogens, and sphingomyelins. Further, we illustrate how groups of metabolites with similar procalcitonin associations form modules which may have relevance to the biological interpretation of our metabolomics observations. Our data suggest that knowledge of such patterns and their biological effects are critical for understanding the role of inflammation in general and procalcitonin specifically.

To put our findings into context, the known properties of highlighted metabolites are discussed as a guide for data interpretation. With increased procalcitonin we find metabolic evidence of mitochondrial dysfunction. We find a specific pattern of change in acylcarnitines with increased procalcitonin where short-chain acylcarnitines are elevated and long-chain acylcarnitines are decreased. Primarily released from the liver, plasma short-chain acylcarnitines (C2–C7) are due to incomplete mitochondrial fatty acid β-oxidation and indicative of impaired mitochondrial function^17^. We also observe an increase in dicarboxylic fatty acids known to be produced by fatty acid omega oxidation when incomplete fatty acid β-oxidation occurs in the setting of mitochondrial dysfunction^18^. The cytochrome P450 (CYP4F) that catalyzes the first step of fatty acid omega oxidation is known to be induced by the pro-inflammatory cytokines IL-1β, IL-6 and TNF-α^19^. Such circulating dicarboxylic fatty acids are shown to increase in response to starvation and critical illness^20^. We additionally note that pentose phosphate pathway metabolites are increased with elevated procalcitonin which suggests a metabolic shift away from fatty acid β-oxidation^21^. Our observation of increases in plasma short-chain acylcarnitines (C2–C7), dicarboxylic fatty acids and pentose phosphate pathway metabolites with increases in procalcitonin may reflect less efficient fatty acid β-oxidation thorough impaired mitochondrial bioenergetics associated with increased inflammation^22^. The mediation of the association between procalcitonin level and metabolite abundance by serum total bilirubin underscores the importance of the liver in metabolism, immunity, inflammation and procalcitonin induction^23^.

Catabolic stress liberates amino acids into the circulation by endogenous protein breakdown including the branched-chain amino acids (BCAA), leucine, isoleucine and valine^24^. During inflammation, BCAAs are preferentially transported to the liver over the muscle^25^. BCAAs are metabolized to acetyl-CoA or succinyl-CoA when mitochondrial fatty acid β-oxidation is incomplete. The irreversible and rate‐limiting step of BCAA catabolism is the branched‐chain α‐ketoacid dehydrogenase (BCKDH) complex in the mitochondrial matrix^26^. Experimental animal data show that BCKDH is rapidly activated by acute nutrient deprivation, circulating BCAA excess, exercise, endotoxin, IL-1β and TNFα^27–31^. Limited evidence suggests that circulating BCAA excess and mitochondrial BCAA catabolism are measurable in healthy humans under exercise stress and also in the critically ill^32–34^.

We observe circulating BCAA catabolic metabolites distal to BCKDH are significantly increased with increasing procalcitonin, suggesting BCKDH activation. Further, we find that short-chain acylcarnitines C3 and C5 are significantly increased with increases in procalcitonin. The C5-acylcarnitines are derived from the BCAA metabolites α-methylbutyryl and isovalerylcarnitine. C3-acylcarnitine is produced from propionyl CoA via catabolism of BCAA, methionine and threonine^35^. Such increases in circulating C5 and C3 acylcarnitines are shown to occur following BCAA supplementation in ambulatory adults^36^. The observed increases of circulating short-chain acylcarnitines, dicarboxylate fatty acids and BCAA catabolic metabolites are all indicative of a metabolic shift. Experimental evidence in healthy humans supports that such a metabolic shift is an adaptive response to endotoxin via alteration of mitochondrial bioenergetics^37^.

We find that higher procalcitonin is associated with increased levels of circulating metabolites of polyamine catabolism**.** Cellular polyamines (spermidine and spermine) are tightly regulated polycations that regulate cell growth and proliferation^38^. Cellular polyamine synthesis is up-regulated during bacterial infections and inflammation^39^. During such inflammatory stress, the catabolic enzyme spermidine/spermine *N*1-acetyltransferase (SSAT) is induced by TNFα. Induced SSAT increases polyamide catabolism and the exit of intracellular polyamine metabolites into the circulation. Such decreases in the concentration of intracellular polyamines lead to slower cell growth rates allowing for potential cell repair or increased apoptosis during inflammation^40^.

Further, we demonstrate that increased procalcitonin is associated with increases in phosphatidylethanolamines and decreases in lysophosphatidylcholine. Phosphatidyl-ethanolamines are present on microparticle surfaces of the endothelium and white blood cells are liberated from endothelial cells following exposure to oxidative stress and found in plasma following experimental sepsis^41,42^. Lysophosphatidylcholines are proinflammatory lipids that activate monocytes, macrophages and T cells. Lower levels of lysophosphatidylcholines are reflective of endothelial dysfunction and are associated with severity of community-acquired pneumonia and sepsis^43–45^. The increased phosphatidylethanolamines and decreased lysophosphatidylcholines with increased procalcitonin observed in our study may reflect endothelial dysfunction from inflammation or direct procalcitonin exposure and indicate dysregulation of the immune response in the setting of more intense inflammation, respectively^9^.

The methodology in our study has multiple strengths. Linear mixed-effects models are vigorous analysis tools for metabolomics studies with repeated time points and multiple clinical variables^46^. Our approach allows for a focus on metabolites that change relative to procalcitonin rather than simply change with the course of critical illness or trial intervention^47^. To limit false positive observations, we conservatively adjusted our mixed-effects significance threshold to account for 983 multiple comparisons. The use of the GGM identification algorithm enhances our association analyses^48^. Further, prior studies show the importance of procalcitonin to the response to severe critical illness which increases the relevance and biological plausibility of our observations^3,7,8,11^.

Our study does have potential limitations. Despite multivariable adjustment, our use of nonrandomized comparisons is subject to bias as subjects with increases in procalcitonin may systematically differ. We performed a post-hoc analysis of plasma samples with correction for multiple testing. Thus, our finding should be considered hypothesis generating. Our study population is heterogenous and increased procalcitonin may be present for different reasons. Further, our study of White critically ill subjects from a single large academic medical center may have limited generalizability. Finally, while the highlighted metabolites have known functional and biological relevance, the clinical significance of a change in metabolite abundance may be unclear.

Taken together, our data indicate that inflammation is associated with alteration of energy utilization by specific metabolic pathways in critical illness. Early critical illness represents a state of nutrient deprivation, oxidative stress and mitochondrial dysfunction which compromise tissue metabolic needs. Circulating metabolites provide an assessment of internal energy states and energy substrate selection. Our findings provide convergent evidence that procalcitonin related inflammation alters mitochondrial bioenergetics. Identifying circulating metabolic information over time is a first step towards understanding the dynamics of energy utilization in critical illness and the metabolomic effects of inflammation.

## Methods

Detailed trial and metabolomics methods are presented in Supplementary Methods. Briefly, the VITdAL-ICU trial (NCT01130181) randomized 475 critically ill adult subjects to vitamin D_3_ or placebo once at a dose of 540,000 IU followed by 90,000 IU monthly^15^. The primary trial outcome was hospital length of stay. Whole blood was collected at randomization (day 0), day 3 and day 7. Frozen plasma was available for analysis in 453 trial subjects. We excluded 9 subjects who did not have serum procalcitonin measured at day 0 and 25 subjects who did not have 25(OH)D measured at day 3.

This study was performed in accordance with the Declaration of Helsinki. At VITdAL-ICU trial enrollment, written informed consent was obtained, if possible, directly from the patient or from a legal surrogate. Consent included permission for plasma specimens to be saved for future research studies. The post-hoc metabolomics study protocol was granted approval by the Mass General Brigham Human Research Committee at the Brigham and Women’s Hospital (Protocol # 2015P002766).

Metabolomics data was generated on a total of 1187 plasma samples from 419 subjects at day 0, 401 subjects at day 3 and 367 subjects at day 7 were analyzed using four ultra high-performance liquid chromatography/tandem accurate mass spectrometry methods by Metabolon, Inc. in 2017^14^. Metabolomic profiling identified 983 plasma metabolites. Individual metabolite raw area count data was normalized, underwent cube root transformation and then Pareto scaling to generate abundance data that were on the same scale and followed an approximate normal distribution.

Our exposure of interest was the individual metabolite abundance. Our primary outcome was serum procalcitonin measured at the same time as the individual metabolite. The procalcitonin level of < 0.5 μg/L was assigned as a cut point indicating severe systemic inflammation^49^. Study sample size was determined utilizing equations for longitudinal studies with a continuous response^50^. We aimed to detect an absolute change in the mean response of procalcitonin of 0.5 μg/L over seven days. We determined the within-subject variability of procalcitonin was 4.1 and the between-subject variability in the rate of change of procalcitonin was 2.0. We utilized an FDR corrected alpha of 0.013, a power of 80%, and three repeated measurements of procalcitonin over seven days. To achieve 80% power, our study requires a study sample of 325 patients.

For univariate analysis of day 0 data, Student’s t test was used to identify metabolites that are associated with a dichotomized procalcitonin measure (procalcitonin < 0.5 μg/L versus procalcitonin ≥ 0.5 μg/L) applying a false discovery rate adjusted p-value (q-value) threshold of 0.05 using MetaboAnalyst^16^. Day 0 data were also analyzed using orthogonal partial least square-discriminant analysis (OPLS-DA), a supervised method to assess the significance of classification discrimination (SIMCA 15.0 Umetrics, Umea, Sweden). We performed permutation testing to validate the OPLS-DA model. We employed sevenfold cross-validation analysis of variance (CV-ANOVA) to determine OPLS-DA model significance. A Receiver Operating Characteristic curve was calculated from class-belonging values predicted by the OPLS-DA model. We produced a misclassification table of the proportion of correctly classified observations (procalcitonin < 0.5 μg/L vs procalcitonin ≥ 0.5 μg/L) in the day 0 data.

For repeated measures data in 419 subjects, the association between relative abundance of individual metabolites (as a continuous exposure) and procalcitonin levels (outcome) at day 0, 3 and 7 were determined utilizing linear mixed-effects models correcting for age, sex, baseline 25(OH)D, absolute increase in 25(OH)D at day 3, SAPS II, plasma day, admission diagnosis and individual subject (as the random-intercept). To identify all significant mixed-effects associations we utilized multiple testing correction based on the Benjamini–Hochberg procedure to adjust the false discovery rate (FDR) to 0.05^51^. All mixed-effects models were analyzed using STATA 16.1MP (College Station, TX). We employed rain plots to visualize effect size and significance relative to procalcitonin levels^52^. Rain plots were produced based on hierarchical clustering in R-3.6.2.

To identify procalcitonin-specific modules from metabolite abundance data, we applied Gaussian graphical models (GGMs) using the metabolomic data from day 0 using the GeneNet R package, version 1.2.13 in R-3.6.2^48^. Modules are identified by reconstruction of pathway reactions derived from metabolomics data. GGMs are determined utilizing partial pairwise Pearson correlation coefficients following the removal of the effects of all other metabolites and covariates^53^. We inferred a procalcitonin-specific network (procalcitonin < 0.5 vs ≥ 0.5 μg/L) for relative metabolite abundance. We included age, sex, SAPS II, admission diagnosis, and baseline 25(OH)D as covariates into the model. Edges between metabolites were allotted if both their Pearson correlations and partial correlations remained statistically significant at a q-value threshold of 0.05^16^.

We finally evaluated a potential mediating effect of bilirubin, age or body mass index on the association between procalcitonin and individual metabolite abundance adjusted for age, sex, baseline 25(OH)D, SAPS II and admission diagnosis. Analyses were performed on each of the 983 metabolites at day 0 using the R package mediation^54^ to obtain bootstrap p-values (N = 2000 samples) for the mediation effect of age or for bilirubin. Significant mediation was present if the p-value was < 0.01 and if ≥ 10% of the association was mediated through bilirubin levels, age or body mass index^14,55^.

## Supplementary Information

Supplementary Legends.

Supplementary Tables.

Dataset S1.

Dataset S2.

Dataset S3.

Dataset S4.

Supplementary Figure 1.

Supplementary Information.

## Data Availability

All data generated or analysed during this study are included in this published article (and its Supplementary Information files).

## References

[1] Maruna P, Nedelnikova K, Gurlich R (2000). Physiology and genetics of procalcitonin. Physiol. Res..

[2] Assicot M (1993). High serum procalcitonin concentrations in patients with sepsis and infection. Lancet.

[3] Christ-Crain M, Muller B (2007). Biomarkers in respiratory tract infections: Diagnostic guides to antibiotic prescription, prognostic markers and mediators. Eur. Respir. J..

[4] Muller B (2001). Ubiquitous expression of the calcitonin-i gene in multiple tissues in response to sepsis. J. Clin. Endocrinol. Metab..

[5] Mitaka C (2005). Clinical laboratory differentiation of infectious versus non-infectious systemic inflammatory response syndrome. Clin. Chim. Acta Int. J. Clin. Chem..

[6] Hu R, Han C, Pei S, Yin M, Chen X (2020). Procalcitonin levels in COVID-19 patients. Int. J. Antimicrob. Agents.

[7] Whang KT (2000). Procalcitonin and proinflammatory cytokine interactions in sepsis. Shock.

[8] Wei JX, Verity A, Garle M, Mahajan R, Wilson V (2008). Examination of the effect of procalcitonin on human leucocytes and the porcine isolated coronary artery. Br. J. Anaesth..

[9] Wagner NM (2017). Procalcitonin impairs endothelial cell function and viability. Anesth. Analg..

[10] Sauer M, Doss S, Ehler J, Mencke T, Wagner NM (2017). Procalcitonin impairs liver cell viability and function in vitro: A potential new mechanism of liver dysfunction and failure during sepsis?. Biomed. Res. Int..

[11] Tavares E, Minano FJ (2010). Immunoneutralization of the aminoprocalcitonin peptide of procalcitonin protects rats from lethal endotoxaemia: Neuroendocrine and systemic studies. Clin. Sci. (Lond.).

[12] Wernerman J (2019). Metabolic support in the critically ill: A consensus of 19. Crit. Care.

[13] Langley RJ (2013). An integrated clinico-metabolomic model improves prediction of death in sepsis. Sci. Transl. Med..

[14] Amrein K, Lasky-Su JA, Dobnig H, Christopher KB (2021). Metabolomic basis for response to high dose vitamin D in critical illness. Clin. Nutr..

[15] Amrein K (2014). Effect of high-dose vitamin D3 on hospital length of stay in critically ill patients with vitamin D deficiency: The VITdAL-ICU randomized clinical trial. JAMA.

[16] Benjamini Y, Hochberg Y (1995). Controlling for false discovery rate: A practical and powerful approach to multiple testing. J. R. Stat. Soc. Ser. B (Methodol.).

[17] Koves TR (2008). Mitochondrial overload and incomplete fatty acid oxidation contribute to skeletal muscle insulin resistance. Cell Metab..

[18] Wanders RJ, Komen J, Kemp S (2011). Fatty acid omega-oxidation as a rescue pathway for fatty acid oxidation disorders in humans. FEBS J..

[19] Kalsotra A (2007). Catalytic characterization and cytokine mediated regulation of cytochrome P450 4Fs in rat hepatocytes. Arch. Biochem. Biophys..

[20] Kemp PR (2020). Metabolic profiling shows pre-existing mitochondrial dysfunction contributes to muscle loss in a model of ICU-acquired weakness. J. Cachexia Sarcopenia Muscle..

[21] Nalos M (2016). Transcriptional reprogramming of metabolic pathways in critically ill patients. Intensive Care Med. Exp..

[22] Vico TA (2019). Mitochondrial bioenergetics links inflammation and cardiac contractility in endotoxemia. Basic Res. Cardiol..

[23] Meisner M, Muller V, Khakpour Z, Toegel E, Redl H (2003). Induction of procalcitonin and proinflammatory cytokines in an anhepatic baboon endotoxin shock model. Shock.

[24] Neinast M, Murashige D, Arany Z (2019). Branched chain amino acids. Annu. Rev. Physiol..

[25] Hasselgren PO, Pedersen P, Sax HC, Warner BW, Fischer JE (1988). Current concepts of protein turnover and amino acid transport in liver and skeletal muscle during sepsis. Arch. Surg..

[26] Harris RA (1990). Regulation of the branched-chain alpha-ketoacid dehydrogenase and elucidation of a molecular basis for maple syrup urine disease. Adv. Enzyme Regul..

[27] Kobayashi R (1999). Hepatic branched-chain alpha-keto acid dehydrogenase complex in female rats: Activation by exercise and starvation. J. Nutr. Sci. Vitaminol. (Tokyo).

[28] Xu M (2001). Mechanism of activation of branched-chain alpha-keto acid dehydrogenase complex by exercise. Biochem. Biophys. Res. Commun..

[29] Shiraki M (2005). Activation of hepatic branched-chain alpha-keto acid dehydrogenase complex by tumor necrosis factor-alpha in rats. Biochem. Biophys. Res. Commun..

[30] Nawabi MD, Block KP, Chakrabarti MC, Buse MG (1990). Administration of endotoxin, tumor necrosis factor, or interleukin 1 to rats activates skeletal muscle branched-chain alpha-keto acid dehydrogenase. J. Clin. Investig..

[31] Rooyackers OE, Senden JM, Soeters PB, Saris WH, Wagenmakers AJ (1995). Prolonged activation of the branched-chain alpha-keto acid dehydrogenase complex in muscle of zymosan treated rats. Eur. J. Clin. Investig..

[32] Overmyer KA (2015). Maximal oxidative capacity during exercise is associated with skeletal muscle fuel selection and dynamic changes in mitochondrial protein acetylation. Cell Metab..

[33] Gamrin L, Essen P, Forsberg AM, Hultman E, Wernerman J (1996). A descriptive study of skeletal muscle metabolism in critically ill patients: Free amino acids, energy-rich phosphates, protein, nucleic acids, fat, water, and electrolytes. Crit. Care Med..

[34] Su L (2015). Dynamic changes in amino acid concentration profiles in patients with sepsis. PLoS One.

[35] Newgard CB (2012). Interplay between lipids and branched-chain amino acids in development of insulin resistance. Cell Metab..

[36] Newgard CB (2009). A branched-chain amino acid-related metabolic signature that differentiates obese and lean humans and contributes to insulin resistance. Cell Metab..

[37] Calvano SE (2005). A network-based analysis of systemic inflammation in humans. Nature.

[38] Mandal S, Mandal A, Johansson HE, Orjalo AV, Park MH (2013). Depletion of cellular polyamines, spermidine and spermine, causes a total arrest in translation and growth in mammalian cells. Proc. Natl. Acad. Sci. U. S. A..

[39] Hardbower DM (2017). Ornithine decarboxylase regulates M1 macrophage activation and mucosal inflammation via histone modifications. Proc. Natl. Acad. Sci. U. S. A..

[40] Babbar N, Murray-Stewart T, Casero RA (2007). Inflammation and polyamine catabolism: The good, the bad and the ugly. Biochem. Soc. Trans..

[41] Colombo S (2018). Phospholipidome of endothelial cells shows a different adaptation response upon oxidative, glycative and lipoxidative stress. Sci. Rep..

[42] Ahn WG, Jung JS, Song DK (2018). Lipidomic analysis of plasma lipids composition changes in septic mice. Korean J. Physiol. Pharmacol..

[43] Arshad H (2019). Decreased plasma phospholipid concentrations and increased acid sphingomyelinase activity are accurate biomarkers for community-acquired pneumonia. J. Transl. Med..

[44] Ahn WG, Jung JS, Kwon HY, Song DK (2017). Alteration of lysophosphatidylcholine-related metabolic parameters in the plasma of mice with experimental sepsis. Inflammation.

[45] van Nieuw Amerongen GP, Vermeer MA, van Hinsbergh VW (2000). Role of RhoA and Rho kinase in lysophosphatidic acid-induced endothelial barrier dysfunction. Arterioscler. Thromb. Vasc. Biol..

[46] Oberg AL, Mahoney DW (2007). Linear mixed effects models. Methods Mol. Biol..

[47] Kelly RS (2017). Integration of metabolomic and transcriptomic networks in pregnant women reveals biological pathways and predictive signatures associated with preeclampsia. Metabolomics.

[48] Do KT (2017). Phenotype-driven identification of modules in a hierarchical map of multifluid metabolic correlations. NPJ Syst. Biol. Appl..

[49] Harbarth S (2001). Diagnostic value of procalcitonin, interleukin-6, and interleukin-8 in critically ill patients admitted with suspected sepsis. Am. J. Respir. Crit. Care Med..

[50] FitzMaurice, G. M., Laird, N. M. & Ware, J. H. *Applied Longitudinal Analysis*. 594–595 (Wiley, 2011).

[51] Benjamini Y, Yekutieli D (2001). The control of the false discovery rate in multiple testing under dependency. Ann. Stat..

[52] Henglin M (2019). A single visualization technique for displaying multiple metabolite-phenotype associations. Metabolites.

[53] Krumsiek J, Suhre K, Illig T, Adamski J, Theis FJ (2011). Gaussian graphical modeling reconstructs pathway reactions from high-throughput metabolomics data. BMC Syst. Biol..

[54] Dustin T, Yamamoto T, Hirose K, Keele L, Imai K (2014). mediation: R package for causal mediation analysis. J. Stat. Softw..

[55] Chary S, Amrein K, Lasky-Su J, Dobnig H, Christopher KB (2021). The sex-specific metabolic response to critical illness: A post-hoc metabolomics study of the VITdAL-ICU trial. Sci. Rep..

