# Supplementary material for: Procalcitonin metabolomics in the critically ill reveal relationships between inflammation intensity and energy utilization pathways
Source: Sci Rep. 2021 Dec 1;11:23194. doi: 10.1038/s41598-021-02679-0 (PMC8636627; doi:10.1038/s41598-021-02679-0)
Supplement: Supplementary file 1 — Supplementary Legends. [file 41598_2021_2679_MOESM1_ESM.pdf]

Supplementary Figure 1. **Unadjusted BCAA metabolite abundance data.** Boxplots with individual data points show relative abundance of plasma BCAAs (A-C), BCKAs (D-F), pre BCKDH metabolites of BCKAs (G-I), metabolites downstream from BCKDH (J-T) and BCAA-derived carnitines (J, K, O, S). Metabolite relative plasma abundance is shown at day 0, 3 and 7 for subjects with procalcitonin<0.5 ug/L or ≥0.5 ug/L. Boxplot indicate 25th (Q1) and 75th (Q3) percentiles, and line through centre of each box represents the median, whiskers indicate minimum (Q1 – 1.5[IQR]) and maximum (Q3 + 1.5[IQR]). Unadjusted metabolite abundance data was normalized in terms of raw area counts and underwent a cube root transformation followed by Pareto scaling. U. Catabolic pathways of BCAAs and involved enzymes (in grey boxes). Metabolites highlighted by circled letters are shown in figures A-Q. Grey circled letters are the metabolite indicated, white circled letters are metabolites of the indicated metabolite (ie 4-methyl-2-oxopentanoate is shown in D and alpha-hydroxyisovalerate a metabolite of 4-methyl-2-oxopentanoate is shown in G). Abbreviations: 3MGH, 3-Methylglutaconyl-Coenzyme-A Hydratase; ACADSB, short/branched chain acyl-CoA dehydrogenase; BCAT, branched chain amino transferase; BCKDH, branched chain amino acid dehydrogenase; HIBDH, 3-hydroxyisobutyrate dehydrogenase; HIBCH, 3-hydroxyisobutyryl-CoA hydrolase; HMG-CoA lyase, 3-hydroxy-3-methylglutaryl-CoA lyase; IBD, Isobutyryl-CoA dehydrogenase; IVD, isovaleryl-CoA dehydrogenase; MCC, methylcrotonoyl-CoA carboxylase; MMSDH, Mammalian Methylmalonate-Semialdehyde Dehydrogenase; SCHMAD: short chain hydroxymethylacyl-CoA dehydrogenase.
