# Supplementary material for: Procalcitonin metabolomics in the critically ill reveal relationships between inflammation intensity and energy utilization pathways
Source: Sci Rep. 2021 Dec 1;11:23194. doi: 10.1038/s41598-021-02679-0 (PMC8636627; doi:10.1038/s41598-021-02679-0)
Supplement: Supplementary file 2 — Supplementary Tables. [file 41598_2021_2679_MOESM2_ESM.pdf]

**Supplemental Tables**

Supplemental Table 1: Additional Cohort Characteristics

Supplemental Table 2: At randomization (Day 0) OPLS-DA model goodness of fit, predictive ability and goodness of fit and predictive ability

Supplemental Table 3: Day 0 Procalcitonin-specific Metabolic Networks with similar effects via Gaussian graphical modeling

## Supplemental Table 1. Additional Cohort Characteristics

| Characteristic | Procalcitonin at Day 0 | | | | | | | | Total | P-value |
|---|---|---|---|---|---|---|---|---|---|---|
| | 0.00 - <0.05 ug/L | 0.05 - <0.10 ug/L | 0.10 - <0.25 ug/L | 0.25 - <0.50 ug/L | 0.50 - <1.00 ug/L | 1.00 - <2.00 ug/L | 2.00 - <10.00 ug/L | ≥10.00 ug/L | | |
| No. | 20 | 42 | 72 | 46 | 69 | 46 | 79 | 45 | 419 | |
| Age years Mean (SD) | 58.9 (15.3) | 61.0 (18.5) | 65.2 (15.1) | 63.8 (12.6) | 66.2 (16.2) | 65.2 (14.4) | 66.4 (13.7) | 62.7 (11.4) | 64.4 (14.8) | 0.27* |
| Female No. (%) | 13 (65) | 18 (43) | 28 (39) | 17 (37) | 23 (33) | 13 (29) | 22 (28) | 15 (33) | 149 (36) | 0.091 |
| SAPS II Mean (SD) | 29.6 (17.0) | 29.7 (14.7) | 35.0 (17.1) | 31.5 (16.7) | 33.2 (14.2) | 34.3 (13.3) | 33.9 (14.4) | 36.1 (16.5) | 33.4 (15.4) | 0.46* |
| C-reactive protein Day 0 Mean (SD) | 22.2 (24.6) | 51.1 (51.1) | 102.7 (70.1) | 123.3 (82.7) | 127.3 (76.2) | 152.4 (83.7) | 163.5 (94.7) | 170.9 (105.0) | 124.2 (90.1) | <0.001* |
| Day 0 25(OH)D Mean (SD) | 14.4 (4.9) | 14.2 (5.1) | 15.0 (6.9) | 14.3 (7.1) | 12.4 (4.1) | 14.1 (6.2) | 14.4 (16.6) | 12.7 (4.8) | 13.9 (8.9) | 0.72* |
| Vitamin $D_3$ Intervention No. (%) | 9 (45) | 21 (50) | 30 (42) | 19 (41) | 39 (57) | 20 (43) | 40 (51) | 28 (62) | 206 (49) | 0.32 |
| Change in 25(OH)D Day 0 to Day 3 Median [IQR] | 2.4 [-0.6, 26.2] | 4.0 [0.1, 43.3] | 2.8 [-0.1, 22.8] | 2.2 [-1.2, 16.1] | 5.4 [0.6, 16.7] | 2.1 [-0.7, 10.9] | 2.5 [0.1, 10.5] | 3.5 [-0.4, 10.1] | 3.1 [0, 16.7] | 0.27† |
| Total Bilirubin Day 0 Mean (SD) | 0.5 (0.3) | 0.6 (0.4) | 0.7 (0.7) | 1.1 (1.5) | 1.1 (1.1) | 1.8 (2.1) | 2.5 (3.0) | 3.6 (5.8) | 1.6 (2.6) | <0.001* |
| Creatinine Day 0 Mean (SD) | 0.7 (0.2) | 0.8 (0.3) | 1.1 (0.8) | 1.1 (0.7) | 1.4 (1.0) | 1.8 (1.0) | 1.7 (1.0) | 2.1 (1.2) | 1.4 (1.0) | <0.001* |
| ICU | | | | | | | | | | |
| Anesthesia ICU No. (%) | 2 (10) | 8 (19) | 10 (14) | 7 (15) | 19 (28) | 10 (22) | 20 (25) | 4 (9) | 80 (19) | |
| Cardiac Surgery ICU No. (%) | 0 (0) | 3 (7) | 14 (19) | 10 (22) | 20 (29) | 20 (43) | 33 (42) | 22 (49) | 122 (29) | |
| Medical ICU No. (%) | 1 (5) | 4 (10) | 12 (17) | 12 (26) | 16 (23) | 11 (24) | 17 (22) | 16 (36) | 89 (21) | |
| Neurological ICU No. (%) | 16 (80) | 27 (64) | 32 (44) | 13 (28) | 8 (12) | 2 (4) | 5 (6) | 3 (7) | 106 (25) | |
| Surgical ICU No. (%) | 1 (5) | 0 (0) | 4 (6) | 4 (9) | 6 (9) | 3 (7) | 4 (5) | 0 (0) | 22 (5) | |
| Admission Diagnosis | | | | | | | | | | |
| Brain Surgery No. (%) | 0 (0) | 1 (2) | 1 (1) | 1 (2) | 0 (0) | 0 (0) | 1 (1) | 0 (0) | 4 (1) | |
| Cardiac surgery No. (%) | 0 (0) | 2 (5) | 9 (13) | 9 (20) | 15 (22) | 11 (24) | 22 (28) | 12 (27) | 80 (19) | |
| Cardiovascular No. (%) | 1 (5) | 1 (2) | 9 (13) | 7 (15) | 9 (13) | 10 (22) | 10 (13) | 3 (7) | 50 (12) | |
| Gastrointestinal/liver No. (%) | 1 (5) | 0 (0) | 0 (0) | 1 (2) | 5 (7) | 3 (7) | 3 (4) | 1 (2) | 14 (3) | |
| Hematologic/oncologic No. (%) | 0 (0) | 0 (0) | 0 (0) | 0 (0) | 1 (1) | 0 (0) | 0 (0) | 0 (0) | 1 (0) | |
| Metabolic No. (%) | 0 (0) | 0 (0) | 0 (0) | 1 (2) | 1 (1) | 0 (0) | 0 (0) | 1 (2) | 3 (1) | |
| Neurologic No. (%) | 16 (80) | 28 (67) | 33 (46) | 10 (22) | 7 (10) | 2 (4) | 6 (8) | 2 (4) | 104 (25) | |
| Other non-operative | 0 (0) | 0 (0) | 1 (1) | 0 (0) | 0 (0) | 0 (0) | 1 (1) | 1 (2) | 3 (1) | |
| Other operative No. (%) | 1 (5) | 1 (2) | 1 (1) | 2 (4) | 3 (4) | 0 (0) | 3 (4) | 1 (2) | 12 (3) | |
| Renal No. (%) | 0 (0) | 0 (0) | 0 (0) | 0 (0) | 2 (3) | 1 (2) | 0 (0) | 2 (4) | 5 (1) | |
| Respiratory No. (%) | 0 (0) | 3 (7) | 6 (8) | 7 (15) | 8 (12) | 3 (7) | 8 (10) | 4 (9) | 39 (9) | |
| Sepsis/infectious No. (%) | 0 (0) | 1 (2) | 3 (4) | 5 (11) | 4 (6) | 3 (7) | 8 (10) | 10 (22) | 34 (8) | |
| Thoracic surgery No. (%) | 0 (0) | 0 (0) | 3 (4) | 0 (0) | 2 (3) | 3 (7) | 4 (5) | 1 (2) | 13 (3) | |
| Transplantation No. (%) | 0 (0) | 0 (0) | 0 (0) | 0 (0) | 0 (0) | 2 (4) | 4 (5) | 6 (13) | 12 (3) | |
| Trauma No. (%) | 1 (5) | 5 (12) | 5 (7) | 3 (7) | 10 (14) | 5 (11) | 6 (8) | 1 (2) | 36 (9) | |
| Vascular surgery No. (%) | 0 (0) | 0 (0) | 1 (1) | 0 (0) | 2 (3) | 3 (7) | 3 (4) | 0 (0) | 9 (2) | |
| 28-day mortality No. (%) | 1 (5) | 4 (10) | 14 (19) | 6 (13) | 11 (16) | 10 (22) | 28 (35) | 21 (47) | 95 (23) | <0.001 |

Data presented as No. (%) unless otherwise indicated. P-values determined by chi-square unless designated by (*) then P-value determined by ANOVA or by (†) determined by Kruskal-Wallis test.

## Supplemental Table 2: At randomization (Day 0) OPLS-DA model goodness of fit and predictive ability

| **OPLS-DA** | | | **Permutation (N=200)** | | **CV-ANOVA** |
|---|---|---|---|---|---|
| R2X | R2Y | Q2 | R2 intercept (*x*-axis, *y*-axis) | Q2 intercept (*x*-axis, *y*-axis) | P-value |
| 0.188 | 0.489 | 0.427 | 0.00, 0.213 | 0.00, -0.214 | <0.001 |

## Supplemental Table 3. Day 0 Procalcitonin-specific Metabolic Networks with similar effects via Gaussian graphical models

| Module | Module q-value | Metabolite | Super Pathway | Sub-pathway | Component q-value |
|---|---|---|---|---|---|
| **A** | **1.11 E-09** | Glutamate | Amino Acid | Glutamate Metabolism | **7.25 E-07** |
| | | cysteine-glutathione disulfide | Amino Acid | Glutathione Metabolism | 4.15 E-01 |
| | | gamma-glutamylglutamine | Peptide | Gamma-glutamyl Amino Acid | **1.93 E-05** |
| | | gamma-glutamylglutamate | Peptide | Gamma-glutamyl Amino Acid | **2.77 E-05** |
| **B** | **1.15 E-05** | Isovalerylglycine | Amino Acid | BCAA Metabolism | **7.03 E-05** |
| | | isobutyrylglycine (C4) | Amino Acid | BCAA Metabolism | **1.44 E-05** |
| | | isobutyrylcarnitine (C4) | Amino Acid | Short-chain Acylcarnitine | **4.04 E-04** |
| **C** | **1.12 E-21** | N-acetylputrescine | Amino Acid | Polyamine Metabolism | **2.58 E-09** |
| | | N-acetyl-beta-alanine | Nucleotide | Pyrimidine Metabolism | **4.21 E-17** |
| | | 4-acetamidobutanoate | Amino Acid | Polyamine Metabolism | **1.84 E-14** |
| | | (N(1) + N(8))-acetylspermidine | Amino Acid | Polyamine Metabolism | **6.94 E-10** |
| | | Acisoga | Amino Acid | Polyamine Metabolism | **7.83 E-12** |
| | | N-acetyl-isoputreanine* | Amino Acid | Polyamine Metabolism | **2.38 E-14** |
| **D** | **1.58 E-20** | N-acetylkynurenine | Amino Acid | Tryptophan Metabolism | **1.12 E-11** |
| | | Kynurenine | Amino Acid | Tryptophan Metabolism | **6.97 E-14** |
| | | N-formylanthranilic acid | Amino Acid | Tryptophan Metabolism | **3.17 E-13** |
| | | N-acetyltryptophan | Amino Acid | Tryptophan Metabolism | **3.63 E-11** |
| | | Quinolinate | Cofactor | Nicotinate and Nicotinamide Metabolism | **1.61 E-14** |
| | | Kynurenate | Amino Acid | Tryptophan Metabolism | **3.39 E-16** |
| **E** | **1.34 E-07** | hexadecenedioate (C16:1-DC)* | Lipid | Fatty Acid, Dicarboxylate | **1.21 E-07** |
| | | hexadecanedioate (C16) | Lipid | Fatty Acid, Dicarboxylate | **1.39 E-06** |
| | | octadecadienedioate (C18:2-DC)* | Lipid | Fatty Acid, Dicarboxylate | **1.45 E-06** |
| | | octadecadienedioate (C18:2-DC)* | Lipid | Fatty Acid, Dicarboxylate | **1.45 E-06** |
| **F** | **4.91 E-07** | glutaroylcarnitine (C5) | Amino Acid | Short-chain Acylcarnitine | **1.49 E-06** |
| | | succinylcarnitine (C4) | Energy | Short-chain Acylcarnitine | **4.74 E-05** |
| **G** | **4.23 E-09** | octanoylcarnitine (C8) | Lipid | Medium-chain Acylcarnitine | **7.10 E-09** |
| | | decanoylcarnitine (C10) | Lipid | Medium-chain Acylcarnitine | **6.22 E-09** |
| **H** | **3.29 E-12** | ximenoylcarnitine (C26:1)* | Lipid | Long-chain Acylcarnitine | **2.21 E-11** |
| | | cerotoylcarnitine (C26)* | Lipid | Long-chain Acylcarnitine | **9.61 E-12** |

Module P-value is the Bonferroni adjusted P-value of the GGM module; Metabolite is the Name of the metabolite in module; Super Pathway is the Name of the major biochemical pathway in the module; Sub-pathway is a subset of the major biochemical pathway in the module. A multiple test-corrected False Discovery Rate threshold of 0.05 was used to identify all significant associations shown in bold. BCAA is Branched-Chain Amino Acids inclusive of Leucine, Isoleucine and Valine. For the Acylcarnitines sub pathway: a capital C is followed by the number of carbons within the fatty acyl group attached to the carnitine. A colon followed by a number is one or more unsaturated carbons in the acylcarnitine ester (i.e. C26:1 is a monounsaturated C26 acylcarnitine). * Putative identification (Level 2) where predictive or externally acquired structure evidence is present when a reference standard does not exist.
