# Supplementary figures and images for: Procalcitonin metabolomics in the critically ill reveal relationships between inflammation intensity and energy utilization pathways
Source: Sci Rep. 2021 Dec 1;11:23194. doi: 10.1038/s41598-021-02679-0 (PMC8636627; doi:10.1038/s41598-021-02679-0)

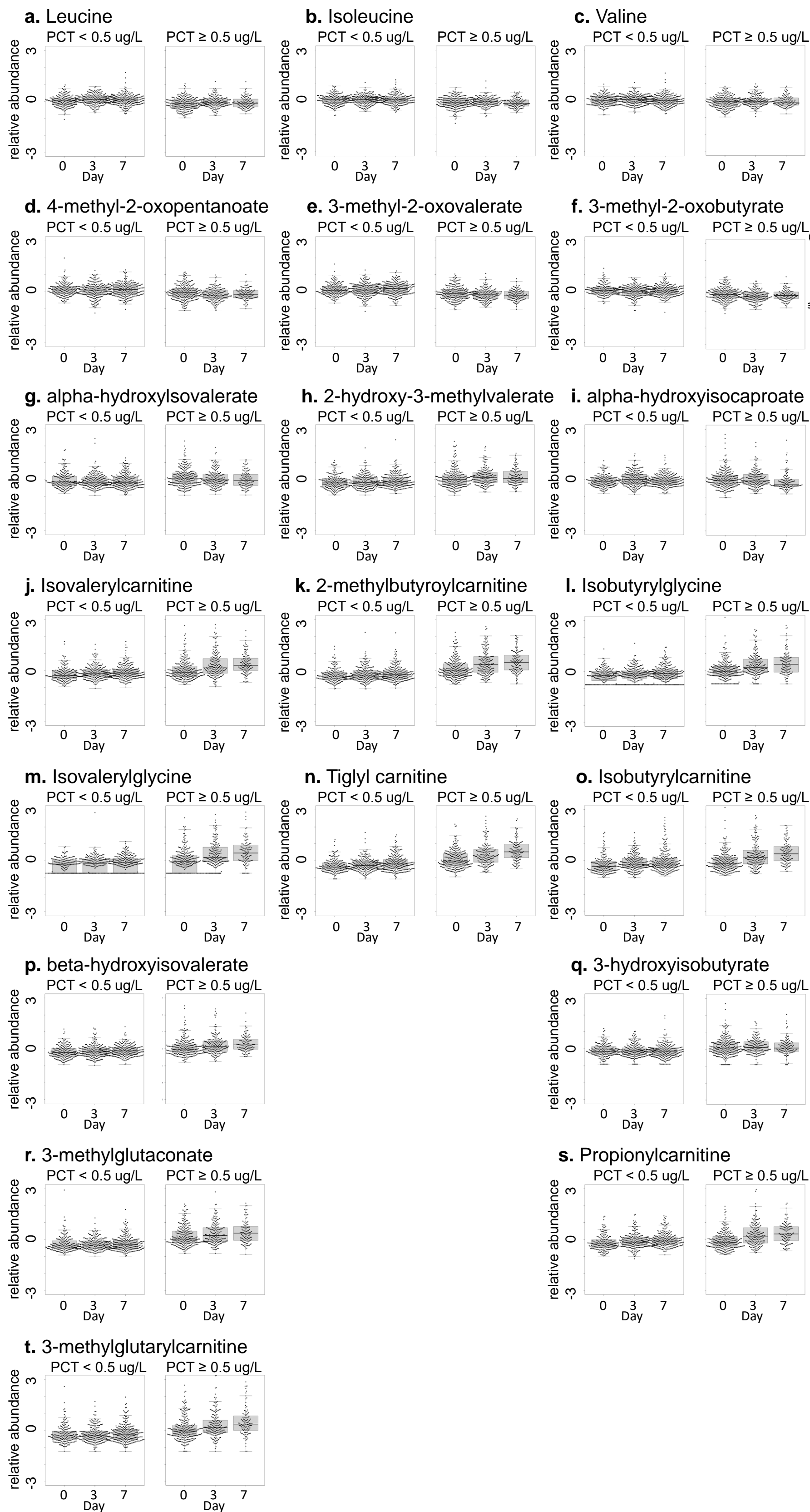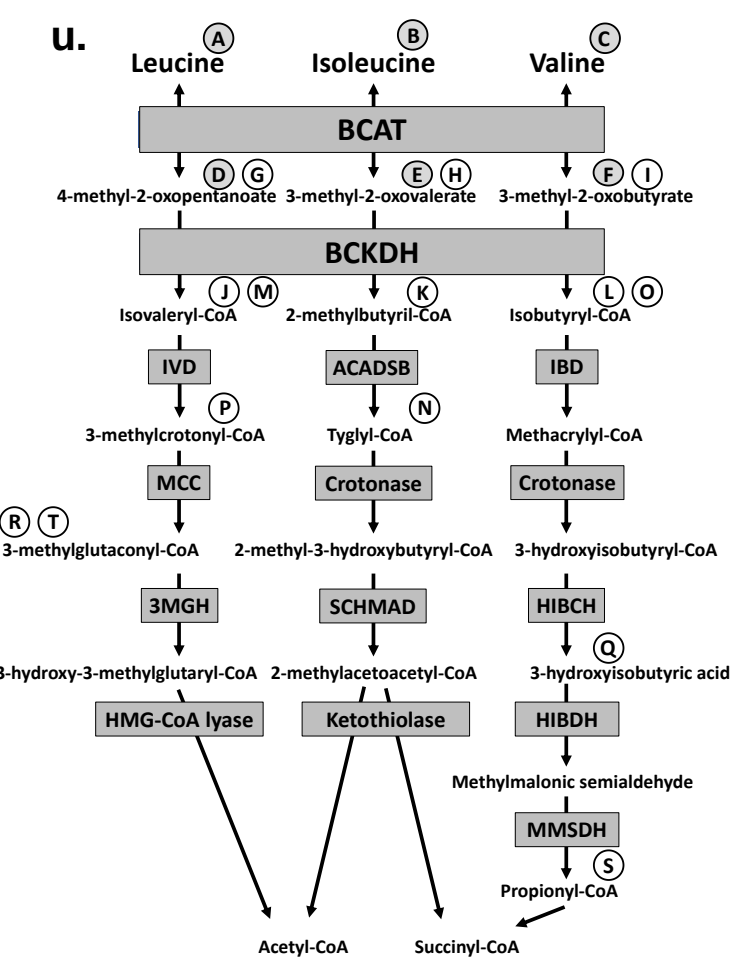

Supplement: Supplementary file 7 — Supplementary Figure 1. [file 41598_2021_2679_MOESM7_ESM.pdf]
