# Supplementary material for: Procalcitonin metabolomics in the critically ill reveal relationships between inflammation intensity and energy utilization pathways
Source: Sci Rep. 2021 Dec 1;11:23194. doi: 10.1038/s41598-021-02679-0 (PMC8636627; doi:10.1038/s41598-021-02679-0)
Supplement: Supplementary file 8 — Supplementary Information. [file 41598_2021_2679_MOESM8_ESM.pdf]

Supplementary Methods

Trial Details: The VITdAL-ICU trial analyzed 475 critically ill adult subjects with 25(OH)D < 20 ng/mL randomized to vitamin D_3 or placebo given orally or via nasogastric tube once at a dose of 540,000 IU followed by 90,000 IU monthly [1]. The trial was conducted at the University Hospital Graz in Southeast Austria in 5 Medical and Surgical Intensive Care Units. Patients were randomized 1:1 with randomization block size of 8 stratified via ICU type and sex. The primary study outcome was length of hospital stay. Secondary outcomes included 28-day mortality, hospital mortality, 6-month mortality, length of ICU stay and 25(OH)D levels at day 0, 3 and 7. Blood samples were collected on days 0 (pre-randomization), 3 and 7. Serum and plasma was fractionated, aliquoted and stored at -70°C. 453 trial subjects had frozen plasma available for analysis. At VITdAL-ICU trial enrollment, written informed consent was obtained, if possible, directly from the patient or from a legal surrogate [1]. Consent included permission for plasma specimens to be saved for future research studies. The post-hoc study research protocol was approved by the Partners Human Research Committee Institutional Review Board at the Brigham and Women's Hospital.

Clinical trial data utilized included age, sex, admission diagnosis category, baseline 25(OH)D, intervention status (placebo vs high dose vitamin D3), absolute change in 25(OH)D level at day 3 relative to day 0, the Simplified Acute Physiology Score (SAPS) II [2] at day 0 and serum procalcitonin at day 0, 3 and 7. Admission diagnosis is determined at ICU admission and categorized by trial investigators and includes Neurosurgery, Cardiac surgery, Cardiovascular, Gastrointestinal/liver,

Hematologic/Oncology/ Metabolic, Neurologic, Other non-operative, Other operative, Renal, Respiratory, Sepsis/infectious, Thoracic surgery, Transplantation, Trauma and Vascular. We excluded 25 trial subjects who did not have serum 25(OH)D measured at day 3 as this data is essential to adjust for intervention response. We excluded 9 subjects who did not have serum procalcitonin measured at day 0.

Study sample size was determined utilizing equations for longitudinal studies with a continuous response [3]. We aimed to detect an absolute change in the mean response of procalcitonin of 0.5 ug/L over seven days. We determined the within-subject variability of procalcitonin, σ_{ϵ}^2 = 4.1 and the between-subject variability in the rate of change of procalcitonin Var(β_{2i})=2. We utilized an FDR corrected alpha of 0.013, a power of 80% (i.e. a=0.013 and γ=0.2), and three repeated measurements of procalcitonin over seven days. Given these conditions

$$\sigma_{\beta}^2 = \sigma_{\epsilon}^2 \left\{ \sum_{j=1}^{n} \left(t_j - \bar{t}\right)^2 \right\}^{-1} + Var(\beta_{2i}) = 2.17$$

The projected total sample size required is

$$N = \frac{\left\{ Z_{\left(1-\alpha/_{2}\right)} + Z_{(1-\gamma)} \right\}^2 4\sigma_{\beta}^2}{\delta^2} = 325$$

Thus, to achieve 80% power, our study requires a study sample of 325 patients.

Sample Preparation: VITdAL-ICU trial subject plasma aliquots were shipped on dry-ice to Metabolon, Inc. Following receipt, the frozen plasma samples were immediately

stored at -80°C. To generate metabolomics data, a total of 1187 plasma samples from 419 subjects at day 0, 401 subjects at day 3 and 367 subjects at day 7 were analyzed using four ultra high-performance liquid chromatography/ tandem accurate mass spectrometry methods by Metabolon, Inc. in 2017 [4]. Plasma sample preparation was performed with the automated MicroLab STAR® Liquid Handling system (Hamilton Company, NV, USA). Before extraction, samples were fortified with recovery standards for quality control (QC) purposes. To remove protein, dissociate small molecules bound to protein or trapped in the precipitated protein matrix, and to recover chemically diverse metabolites, proteins were precipitated with methanol via 2 minutes of robust shaking (GenoGrinder 2000 SPEX SamplePrep, NJ, USA) and subsequent centrifugation. The resulting extract was divided into five fractions: two for analysis by two separate reverse phase (RP)/UPLC-MS/MS methods with positive ion mode electrospray ionization (ESI), one for analysis by RP/UPLC-MS/MS with negative ion mode ESI, one for analysis by HILIC/UPLC-MS/MS with negative ion mode ESI, and one sample was reserved for backup. Samples were placed on a TurboVap® (Zymark, MA, USA) to remove the organic solvent and stored overnight under nitrogen before preparation for analysis.

Quality Assurance (QA) and Quality Control (QC): Several types of controls were utilized with the plasma samples analysis: a pooled matrix sample generated by taking a small volume of each experimental sample served as a technical replicate throughout the data set [5]; extracted water samples served as process blanks [6]; and a cocktail of QC standards that were carefully chosen not to interfere with the measurement of endogenous compounds were spiked into every analyzed sample [7], allowed instrument

performance monitoring and aided chromatographic alignment. Instrument variability was determined by calculating the median relative standard deviation (RSD) for the standards that were added to each sample prior to injection into the mass spectrometers [8]. Overall process variability was determined by calculating the median RSD for all endogenous metabolites (i.e., non-instrument standards) present in 100% of the pooled matrix samples. Experimental samples were randomized across the platform run with QC samples spaced evenly among the injections.

Ultrahigh Performance Liquid Chromatography-Tandem Mass Spectroscopy (UPLC-MS/MS): All methods utilized a Waters ACQUITY ultra-performance liquid chromatography (UPLC) (Waters, MA, USA) and for untargeted lipidomic analysis a Thermo Scientific Q Exactive™ high resolution/accurate mass spectrometer interfaced with a heated electrospray ionization (HESI-II) source and Orbitrap™ mass analyzer operated at 35,000 mass resolution (ThermoFisher Scientific, MA, USA) [9]. The sample extract was dried then reconstituted in solvents compatible to each of the four methods. Each reconstitution solvent contained a series of standards at fixed concentrations to ensure injection and chromatographic consistency. One aliquot was analyzed using acidic positive ion conditions, chromatographically optimized for more hydrophilic compounds. In this method, the extract was gradient eluted from a C18 column (Waters UPLC BEH C18-2.1x100 mm, 1.7 µm) using water and methanol, containing 0.05% perfluoropentanoic acid (PFPA) and 0.1% formic acid (FA). Another aliquot was also analyzed using acidic positive ion conditions, however it was chromatographically optimized for more hydrophobic compounds [10,11]. In this method, the extract was

gradient eluted from the same afore mentioned C18 column using methanol, acetonitrile, water, 0.05% PFPA and 0.01% FA and was operated at an overall higher organic content. Another aliquot was analyzed using basic negative ion optimized conditions using a separate dedicated C18 column. The basic extracts were gradient eluted from the column using methanol and water, however with 6.5mM Ammonium Bicarbonate at pH 8. The fourth aliquot was analyzed via negative ionization following elution from a HILIC column (Waters UPLC BEH Amide 2.1x150 mm, 1.7 µm) using a gradient consisting of water and acetonitrile with 10mM Ammonium Formate, pH 10.8. The MS analysis alternated between MS and data-dependent MS^n scans using dynamic exclusion [12]. The scan range for both ionization modes was 70–1000 *m/z* [13].

Data Extraction and Compound Identification: Raw data was extracted, peak-identified and QC processed using Metabolon's hardware and software. Compounds were identified by comparison to library entries of purified standards or recurrent unknown entities. Metabolon maintains a library based on authenticated standards that contains the retention time/index (RI), mass-to-charge ratio (*m/z*), and chromatographic data (including MS/MS spectral data) on all molecules present in the library. Furthermore, biochemical identifications are based on three criteria: retention index within a narrow RI window of the proposed identification, accurate mass match to the library +/- 10 ppm, and the MS/MS forward and reverse scores between the experimental data and authentic standards [14]. The MS/MS scores are based on a comparison of the ions present in the experimental spectrum to the ions present in the library spectrum. While there may be similarities between these molecules based on

one of these factors, the use of all three data points can be utilized to distinguish and differentiate biochemicals [15]. More than 3300 commercially available purified standard compounds have been acquired and registered into the Metabolon Laboratory Information Management System (LIMS) system for analysis on all platforms for determination of their analytical characteristics. The identification level reported in our tables follows the criteria described by Sumner et al. [16]. Level 1 is a validated identification which confirms a structure with a minimum of two independent and orthogonal data from a pure reference standard under identical analytical conditions. Predictive or externally acquired structure evidence when a reference standard does not exist, (i.e. MS/MS data, exhibiting diagnostic fragments or neutral losses consistent with a specific structure) is a putative identification (Level 2) [17]. Compounds labelled with "*" have identification Level 2. If no label is applied, the identification Level is 1. Compounds labelled with "( )" or "[ ]" indicate a structural isomer of another compound in the spectral library; for example, a steroid that may be sulfated at one of several positions that are indistinguishable by the mass spectrometry data or a diacylglycerol for which more than one stereospecific molecule exists. For the Acylcarnitine sub pathway: a capital C is followed by the number of carbons within the fatty acyl group attached to the carnitine. A colon followed by a number is one or more unsaturated carbons in the acylcarnitine ester (i.e. C10:1 is a monounsaturated C10 acylcarnitine). DC following the carbon number is a dicarboxylic acylcarnitine. Acylcarnitines are classified by the number of carbon atoms in the acyl group chain: short-chain acylcarnitines C2 to C7; medium-chain acylcarnitines C8 to C14; long-chain acylcarnitines C16 – C26 [18]. A summary of all 983 metabolites identified is present in Supplementary Data 5.

Curation: A variety of curation procedures were carried out to ensure that a high quality data set was made available for statistical analysis and data interpretation. The QC and curation processes were designed to ensure accurate and consistent identification of true chemical entities, and to remove those representing system artifacts, mis-assignments, and background noise. Metabolon data analysts use proprietary visualization and interpretation software to confirm the consistency of peak identification among the various samples. Library matches for each compound were checked for each sample and corrected if necessary.

Metabolite Quantification and Data Normalization: Peaks were quantified using total spectral area (area under the curve) [19-21]. Metabolite quantitation or abundance is defined as the total ion count for the given mass-to-charge ratio (*m/z*) assigned to the particular metabolite (Supplementary Data 5) [22]. Specifically, metabolite quantitation is determined using extracted ion chromatograms by focusing the narrow mass window on the theoretical *m/z* value of the individual metabolite of interest and eliminating overlapping isobaric signals with maintenance of the mass accuracy during the acquisition [23-27]. A data normalization step was performed to correct variation resulting from instrument inter-day tuning differences. Each compound was corrected in run-day blocks by registering the medians to equal one (1.00) and normalizing each data point proportionately.

Statistical Analysis: Determination of the changes in relative concentrations of metabolites was first suggested as a strategy to define the metabolome in 1998 [28]. We employed metabolomic profiling to identify 983 metabolites (Supplementary Data 5). Metabolomic data underwent a cube root transformation followed by Pareto scaling to generate data that were on the same scale and followed an approximate normal distribution [29,30].

Our exposure of interest was the individual metabolite abundance. Our primary outcome was serum procalcitonin measured at the same time as the individual metabolite. The procalcitonin level of <0.5 ug/L was assigned as a cut point indicating severe systemic inflammation [31]. For univariate analysis of day 0 data, Student's t test was used to determine the significance of each metabolite between procalcitonin < 0.5 ug/L versus procalcitonin ≥ 0.5 ug/L groups using MetaboAnalyst [32]. To identify all significant associations we utilized multiple testing correction based on the Benjamini-Hochberg procedure to adjust the false-discovery rate (FDR) to 0.05 [33]. Day 0 data was also analyzed using orthogonal partial least square-discriminant analysis (OPLS-DA), also known as orthogonal projections to latent structures discriminant analysis, a supervised method to assess the significance of classification discrimination (SIMCA 15.0 Umetrics, Umea, Sweden) [34-36]. OPLS-DA was performed to relate the X data to the Y response [35,37]. In our study, the X are the metabolites at day 0 and the Y is the intervention (procalcitonin < 0.5 ug/L versus procalcitonin ≥ 0.5 ug/L groups). We assessed the OPLS-DA model quality via the variation of X explained by the model (R2X(cum)); the goodness-of-fit represented by the percentage of the variation of Y explained by the

model (R2); and the predictive performance (Q2). Permutation testing was performed to validate the OPLS-DA model [38,39]. The percentage of the variation of the dataset predicted by the model (Permuted Q2) was assessed using a cross-validation test [40,41]. Sevenfold cross-validation analysis of variance (CV-ANOVA) was utilized to determine OPLS-DA model significance [39]. Additionally, response permutation testing was performed to validate the OPLS-DA model [38,39]. To this end, the intervention is permutated to appear in a different order while the metabolite-dataset remains intact. Next, a model is then fit to the permutated data. The goodness-of-fit (R2) and predictive performance (Q2) of the permutated model are contrasted to the actual model. A valid model has lower permutated Q2 values compared to the actual model and the Q2-intercept is below zero. A Receiver Operating Characteristic (ROC) curve was calculated from class-belonging values predicted by the OPLS-DA model. We produced a misclassification table of the proportion of correctly classified observations (procalcitonin < 0.5 ug/L vs procalcitonin ≥ 0.5 ug/L) in the day 0 data [42].

For repeated measures data, the association between relative abundance of individual metabolites (outcome) and procalcitonin levels (as a continuous exposure) at day 0, 3 and 7 were determined utilizing linear mixed-effects regression models correcting for age, sex, baseline 25(OH)D, absolute increase in 25(OH)D at day 3, SAPS II, plasma day, admission diagnosis and individual subject (as the random-intercept). A total of 1187 plasma samples from 419 subjects at day 0, 401 subjects at day 3 and 367 subjects at day 7 were analyzed with linear mixed-effects models. We fit the linear mixed effects model to the procalcitonin determined on day 0, 3 or 7, as measured on a

continuous scale for improved predictive performance [43]. A false discovery rate adjusted p-value (q-value) threshold of 0.05 was used to identify all significant mixed-effects associations [44]. All mixed-effects models were analyzed using STATA 14.1MP (College Station, TX). We employed rain plots [45] to visualize effect size, significance, clustering and trends across procalcitonin levels. Rain plots were produced in R-3.6.2 adapted from source code published by Henglin et.al. [45].

To identify procalcitonin-specific modules from metabolite abundance data, we estimated Gaussian graphical models (GGMs) for day 0 using the GeneNet R package, version 1.2.13 in R-3.6.2 adapted from source code published by Do et.al. [46]. Modules are identified by reconstruction of pathway reactions derived from metabolomics data. GGMs are determined utilizing partial pairwise Pearson correlation coefficients following the removal of the effects of all other metabolites and covariates [47]. GGMs are representations of the linear association between two metabolites corrected for other confounding variables in multivariate Gaussian distributions. We inferred a procalcitonin-specific network (procalcitonin <0.05 vs ≥ 0.05 ug/L) for relative metabolite abundance. We included age, sex, SAPS II, admission diagnosis and baseline 25(OH)D as covariates into the model. We allocated edges between metabolites if both their Pearson correlations and partial correlations remained statistically significant at a q-value threshold of 0.05 [48].

As the liver is a lymphoid organ [49], age is an important regulator of inflammation [50], and procalcitonin levels are associated with obesity [51] we evaluated a potential mediating effect of bilirubin, age or body mass index on the association between procalcitonin and individual metabolite abundance adjusted for age, sex, baseline 25(OH)D, absolute increase in 25(OH)D, SAPS II and admission diagnosis. Analyses were performed on each of the 983 metabolites at day 0 using the R package mediation [52] to obtain bootstrap P values (N = 2000 samples) for the mediation effect of age or for bilirubin. Significant mediation was present if the p-value was < 0.01 and if ≥10% of the association was mediated through bilirubin levels, age or body mass index [53,54].

Supplemental Methods References Cited

1 Amrein, K. *et al.* Effect of high-dose vitamin D3 on hospital length of stay in critically ill patients with vitamin D deficiency: the VITdAL-ICU randomized clinical trial. *JAMA* **312**, 1520-1530, doi:10.1001/jama.2014.13204 (2014).

2 Le Gall, J. R., Lemeshow, S. & Saulnier, F. A new Simplified Acute Physiology Score (SAPS II) based on a European/North American multicenter study. *JAMA* **270**, 2957-2963, doi:10.1001/jama.270.24.2957 (1993).

3 FitzMaurice, G. M., Laird, N. M. & Ware, J. H. *Applied longitudinal analysis*. 594-595 (Wiley, 2011).

4 Amrein, K., Lasky-Su, J. A., Dobnig, H. & Christopher, K. B. Metabolomic basis for response to high dose vitamin D in critical illness. *Clinical nutrition*, doi:10.1016/j.clnu.2020.09.028 (2020).

5 Wehrens, R. *et al.* Improved batch correction in untargeted MS-based metabolomics. *Metabolomics* **12**, 88, doi:10.1007/s11306-016-1015-8 (2016).

6 Trezzi, J. P. *et al.* Metabolic profiling of body fluids and multivariate data analysis. *MethodsX* **4**, 95-103, doi:10.1016/j.mex.2017.02.004 (2017).

7 Bain, J. R. *et al.* Metabolomics applied to diabetes research: moving from information to knowledge. *Diabetes* **58**, 2429-2443, doi:10.2337/db09-0580 (2009).

8 Parsons, H. M., Ekman, D. R., Collette, T. W. & Viant, M. R. Spectral relative standard deviation: a practical benchmark in metabolomics. *Analyst* **134**, 478-485, doi:10.1039/b808986h (2009).

9 Narvaez-Rivas, M. & Zhang, Q. Comprehensive untargeted lipidomic analysis using core-shell C30 particle column and high field orbitrap mass spectrometer. *J Chromatogr A* **1440**, 123-134, doi:10.1016/j.chroma.2016.02.054 (2016).

10 Michopoulos, F., Lai, L., Gika, H., Theodoridis, G. & Wilson, I. UPLC-MS-based analysis of human plasma for metabonomics using solvent precipitation or solid phase extraction. *J Proteome Res* **8**, 2114-2121, doi:10.1021/pr801045q (2009).

11 Want, E. J., Smith, C. A., Qin, C., Van Horne, K. C. & Siuzdak, G. Phospholipid capture combined with non-linear chromatographic correction for improved serum metabolite profiling. *Metabolomics* **2**, 145-154 (2006).

12 Oresic, M., Vidal-Puig, A. & Hanninen, V. Metabolomic approaches to phenotype characterization and applications to complex diseases. *Expert Rev Mol Diagn* **6**, 575-585, doi:10.1586/14737159.6.4.575 (2006).

13 Chen, W. W., Freinkman, E., Wang, T., Birsoy, K. & Sabatini, D. M. Absolute Quantification of Matrix Metabolites Reveals the Dynamics of Mitochondrial Metabolism. *Cell* **166**, 1324-1337 e1311, doi:10.1016/j.cell.2016.07.040 (2016).

14 Hufsky, F., Scheubert, K. & Bocker, S. Computational mass spectrometry for small-molecule fragmentation. *TrAC Trends Anal. Chem.* **53**, 41–48 (2014).

15 Dunn, W. B. *et al.* Procedures for large-scale metabolic profiling of serum and plasma using gas chromatography and liquid chromatography coupled to mass spectrometry. *Nat Protoc* **6**, 1060-1083, doi:10.1038/nprot.2011.335 (2011).

16 Sumner, L. W. *et al.* Proposed minimum reporting standards for chemical analysis Chemical Analysis Working Group (CAWG) Metabolomics Standards

Initiative (MSI). *Metabolomics* **3**, 211-221, doi:10.1007/s11306-007-0082-2 (2007).
17 Schrimpe-Rutledge, A. C., Codreanu, S. G., Sherrod, S. D. & McLean, J. A. Untargeted Metabolomics Strategies-Challenges and Emerging Directions. *J Am Soc Mass Spectrom* **27**, 1897-1905, doi:10.1007/s13361-016-1469-y (2016).
18 Guasch-Ferre, M. *et al.* Plasma acylcarnitines and risk of cardiovascular disease: effect of Mediterranean diet interventions. *The American journal of clinical nutrition* **103**, 1408-1416, doi:10.3945/ajcn.116.130492 (2016).
19 Weljie, A. M., Newton, J., Mercier, P., Carlson, E. & Slupsky, C. M. Targeted profiling: quantitative analysis of 1H NMR metabolomics data. *Anal Chem* **78**, 4430-4442, doi:10.1021/ac060209g (2006).
20 Wishart, D. S. Quantitative metabolomics using NMR. *TrAC Trends Anal. Chem.* **27**, 228–237 (2008).
21 Zhou, B., Xiao, J. F., Tuli, L. & Ressom, H. W. LC-MS-based metabolomics. *Mol Biosyst* **8**, 470-481, doi:10.1039/c1mb05350g (2012).
22 Parisi, L. R., Li, N. & Atilla-Gokcumen, G. E. Very Long Chain Fatty Acids Are Functionally Involved in Necroptosis. *Cell Chem Biol* **24**, 1445-1454 e1448, doi:10.1016/j.chembiol.2017.08.026 (2017).
23 Junot, C., Madalinski, G., Tabet, J. C. & Ezan, E. Fourier transform mass spectrometry for metabolome analysis. *Analyst* **135**, 2203-2219, doi:10.1039/c0an00021c (2010).
24 Kamleh, A. *et al.* Metabolomic profiling using Orbitrap Fourier transform mass spectrometry with hydrophilic interaction chromatography: a method with wide applicability to analysis of biomolecules. *Rapid Commun Mass Spectrom* **22**, 1912-1918, doi:10.1002/rcm.3564 (2008).
25 Kamleh, M. A., Hobani, Y., Dow, J. A. & Watson, D. G. Metabolomic profiling of Drosophila using liquid chromatography Fourier transform mass spectrometry. *FEBS Lett* **582**, 2916-2922, doi:10.1016/j.febslet.2008.07.029 (2008).
26 Koulman, A. *et al.* High-resolution extracted ion chromatography, a new tool for metabolomics and lipidomics using a second-generation orbitrap mass spectrometer. *Rapid Commun Mass Spectrom* **23**, 1411-1418, doi:10.1002/rcm.4015 (2009).
27 Xiao, J. F., Zhou, B. & Ressom, H. W. Metabolite identification and quantitation in LC-MS/MS-based metabolomics. *Trends Analyt Chem* **32**, 1-14, doi:10.1016/j.trac.2011.08.009 (2012).
28 Oliver, S. G., Winson, M. K., Kell, D. B. & Baganz, F. Systematic functional analysis of the yeast genome. *Trends Biotechnol* **16**, 373-378, doi:10.1016/s0167-7799(98)01214-1 (1998).
29 van den Berg, R. A., Hoefsloot, H. C., Westerhuis, J. A., Smilde, A. K. & van der Werf, M. J. Centering, scaling, and transformations: improving the biological information content of metabolomics data. *BMC Genomics* **7**, 142, doi:10.1186/1471-2164-7-142 (2006).
30 Struja, T. *et al.* Metabolomics for Prediction of Relapse in Graves' Disease: Observational Pilot Study. *Front Endocrinol (Lausanne)* **9**, 623, doi:10.3389/fendo.2018.00623 (2018).

31 Harbarth, S. *et al.* Diagnostic value of procalcitonin, interleukin-6, and interleukin-8 in critically ill patients admitted with suspected sepsis. *Am J Respir Crit Care Med* **164**, 396-402, doi:10.1164/ajrccm.164.3.2009052 (2001).
32 Chong, J. & Xia, J. Using MetaboAnalyst 4.0 for Metabolomics Data Analysis, Interpretation, and Integration with Other Omics Data. *Methods Mol Biol* **2104**, 337-360, doi:10.1007/978-1-0716-0239-3_17 (2020).
33 Benjamini, Y. & Yekutieli, D. The control of the false discovery rate in multiple testing under dependency. *Ann Stat* **29**, 1165-1188 (2001).
34 Trygg, J. O2-PLS for qualitative and quantitative analysis in multivariate calibration. *J. Chemometrics* **16**, 283-293 (2002).
35 Trygg, J. & Wold, S. Orthogonal projections to latent structures (O-PLS). *J. Chemometrics* **16**, 119–128 (2002).
36 Trygg, J. & Wold, S. O2-PLS, a two block (X – Y) latent variable regression (LVR) method with an integral OSC filter. *J. Chemometrics* **17** (2003).
37 Bylesjö, M. *et al.* OPLS discriminant analysis: combining the strengths of PLS-DA and SIMCA classification. *Journal of Chemometrics* **20**, 341–351 (2006).
38 Westerhuis, J. A. *et al.* Assessment of PLSDA cross validation. *Metabolomics* **4**, 81–89 (2008).
39 Eriksson, L., Trygg, J. & Wold, S. CV-ANOVA for significance testing of PLS and OPLS models. *Journal of Chemometrics* **22**, 594-600 (2008).
40 Eastment, H. & Krzanowski, W. Crossvalidatory choice of the number of components from a principal component analysis. *Technometrics* **24**, 73-77 (1982).
41 Martens, H. & Naes, T. *Multivariate Calibration*. (John Wiley and Sons, 1989).
42 Blasco, H. *et al.* Comparative analysis of targeted metabolomics: dominance-based rough set approach versus orthogonal partial least square-discriminant analysis. *J Biomed Inform* **53**, 291-299, doi:10.1016/j.jbi.2014.12.001 (2015).
43 Foulkes, A. S. *et al.* Prediction based classification for longitudinal biomarkers. *Ann Appl Stat* **4**, 1476-1497, doi:10.1214/10-AOAS326 (2010).
44 Storey, J. D. & Tibshirani, R. Statistical significance for genomewide studies. *Proc Natl Acad Sci U S A* **100**, 9440-9445, doi:10.1073/pnas.1530509100 (2003).
45 Henglin, M. *et al.* A Single Visualization Technique for Displaying Multiple Metabolite-Phenotype Associations. *Metabolites* **9**, doi:10.3390/metabo9070128 (2019).
46 Do, K. T. *et al.* Phenotype-driven identification of modules in a hierarchical map of multifluid metabolic correlations. *NPJ Syst Biol Appl* **3**, 28, doi:10.1038/s41540-017-0029-9 (2017).
47 Krumsiek, J., Suhre, K., Illig, T., Adamski, J. & Theis, F. J. Gaussian graphical modeling reconstructs pathway reactions from high-throughput metabolomics data. *BMC Syst Biol* **5**, 21, doi:10.1186/1752-0509-5-21 (2011).
48 Benjamini, Y. & Hochberg, Y. Controlling for false discovery rate: a practical and powerful approach to multiple testing. *Journal of the Royal Statistical Society. Series B (Methodological)* **57**, 289–300 (1995).
49 Crispe, I. N. The liver as a lymphoid organ. *Annu Rev Immunol* **27**, 147-163, doi:10.1146/annurev.immunol.021908.132629 (2009).

50 Albright, J. M. *et al.* Advanced Age Alters Monocyte and Macrophage Responses. *Antioxid Redox Signal* **25**, 805-815, doi:10.1089/ars.2016.6691 (2016).

51 Abbasi, A. *et al.* Plasma procalcitonin is associated with obesity, insulin resistance, and the metabolic syndrome. *J Clin Endocrinol Metab* **95**, E26-31, doi:10.1210/jc.2010-0305 (2010).

52 Dustin, T., Yamamoto, T., Hirose, K., Keele, L. & Imai, K. mediation: R package for causal mediation analysis. *Journal of Statistical Software* **59**, 1-38 (2014).

53 Masuch, A. *et al.* Metabolomic profiling implicates adiponectin as mediator of a favorable lipoprotein profile associated with NT-proBNP. *Cardiovasc Diabetol* **17**, 120, doi:10.1186/s12933-018-0765-1 (2018).

54 Pietzner, M. *et al.* Hepatic Steatosis Is Associated With Adverse Molecular Signatures in Subjects Without Diabetes. *J Clin Endocrinol Metab* **103**, 3856-3868, doi:10.1210/jc.2018-00999 (2018).
